# Design, development, in-vitro and in-vivo evaluation of polylactic acid-based multifunctional nanofibrous patches for efficient healing of diabetic wounds

**DOI:** 10.1038/s41598-023-29032-x

**Published:** 2023-02-24

**Authors:** Isra H. Ali, Islam A. Khalil, Ibrahim M. El-Sherbiny

**Affiliations:** 1 Nanomedicine Research Labs, Center for Materials Science (CMS), Zewail City of Science and Technology, 6th of October City, Giza, 12578 Egypt; 2 Department of Pharmaceutics, College of Pharmacy and Drug Manufacturing, Misr University of Science and Technology (MUST), 6th of October City, Giza, 12566 Egypt

**Keywords:** Biochemistry, Materials science, Nanoscience and technology

## Abstract

Impaired healing of diabetic ulcers is one of the major complications of diabetic patients due to high susceptibility to microbial infections, impaired lymphianogenesis, edema, and consequently impairing proper healing. This could even lead to much worse complications that include severe gangrene, trauma and finally limb amputation. Therefore, this study aims to develop a multilayered durable nanofibrous wound patch loaded with three promising drugs (phenytoin, sildenafil citrate and simvastatin) each in a separate layer to target a different wound healing phase. Polylactic acid was used for the preparation of the nanofibrous matrix of the wound patch, where each drug was incorporated in a separate layer during the preparation process. Drugs release profiles were studied over 3 weeks. Results showed that both phenytoin and simvastatin were released within 14 days while sildenafil continued till 21 days. Both physicochemical and mechanical characteristics of the patches were fully assessed as well as their biodegradability, swellability, breathability and porosity. Results showed that incorporation of drugs preserved the physicochemical and mechanical properties as well as porosity of the developed nanofibers. In addition, patches were evaluated for their biocompatibility and cell adhesion capability before being tested through in-vivo diabetic wound rat model induced by alloxan for three weeks. In vivo results showed that the patches were successful in inducing proper wound healing in diabetic rat model with overcoming the above-mentioned obstacles within 3 weeks. This was confirmed through assessing wound closure as well as from histopathological studies that showed complete healing with proper cell regeneration and arrangement without forming scars.

## Introduction

Diabetes mellitus is one of the vast spreading metabolic disorders worldwide. According to the International Diabetes Federation (IDF), more than 400 million adults worldwide are suffering from diabetes. And they are expected reach 600 million patients by 2045^1^.

Diabetic ulcer is one of the major diabetic complications. Briefly, slow rate of ulcer healing leads to microbial infection, gangrene, and trauma. Infected foot ulcer usually leads to hospitalization and ends up with amputation^2–4^. Delayed healing in diabetic patients is attributed to complicated pathological features that retard the healing process^5^. These include: (1) increased apoptosis, alkalinity, inflammation and edema, (2) impaired lymphianogenesis^6,7^, (3) delayed angiogenesis^8,9^, (4) reduced collagen deposition^10,11^, and (5) reduced rate of fibroblasts and myofibroblasts proliferation^12–14^.

Although wound closure occurs naturally, it is extremely complicated in diabetic patients as there is no well-defined followed protocol till now for the complete healing of diabetic wounds and ulcers^2,15^.

Wound healing is a complex process that involves extracellular matrices (ECM), red and white blood cells, platelets, growth factors, cytokines and other factors^16^. Although some wounds appear to heal naturally through the normal phenomenon of skin restoration, perfect tissue regeneration and re-organization of cells are hard to be achieved especially in complicated wounds like diabetic ulcer^6,17,18^.

Wound healing involves four overlapping phases that occur successively. The initial (first) phase involves the hemostatic role forming blood clots within the wound cut. It takes the shortest duration which extends from few minutes after the injury to few hours. Then, the second phase that is responsible for the defensive role starts after few hours and extends to one week approximately. It involves migration of both leukocytes (neutrophils) and monocytes from blood vessels into damaged tissues, and differentiating into macrophages.

In addition, both angiogenesis and lymphianogenesis start during that stage. After around 5 days of the injury, the third phase comes to extend through the following 10–14 days. During that stage, both angiogenesis and lymphianogenesis continue to aid the process of proliferation of fibroblasts, myofibroblasts and keratinocytes to form the dermal and epidermal layers, respectively. Finally, the final (fourth) phase completes the healing process through stimulating collagen deposition and connective tissues formation fibroblasts and keratinocytes layers, respectively leading to wound contraction and closure^16,19^.

Therefore, using traditional commercial (passive) wound dressings for diabetic wounds is not sufficient, since these dressings act only as a protection layer against microbial infection. However, they do not have any bioactive materials or drugs to aid in wound treatment or stimulate wound healing phases in chronic or complicated wounds especially in diabetic patients. On the other hand, active wound dressings that contain bioactive materials such as drugs, growth factors and cells were found to be more efficient in cell restoration and proper healing of chronic wounds^20,21^. Therefore, developing multi-functional wound dressings possessing highly porous structures that can mimic the extracellular matrices of skin layers is a mandatory step in order to help in stimulating the different phases of wound healing process^21–25^.

Nanofibers (NFs) have been developed using several techniques such as phase separation, self-assembly, solvent casting, drawing, gas foaming, solid-free forming, freeze drying, template synthesis, melt molding, and particulate leaching. However, all of these techniques were reported for their failure to produce NFs with controlled porosity and other physical properties^18,26–28^. Electrospinning, however was reported for its high reliability to produce NFs with controlled features such as porosity and dimensions when compared to other traditional techniques. This may be attributed to the high efficiency of electrospinner to develop interconnected meshes with controlled features^29–31^. Hence, electrospinning, a promising technique to fabricate uniform and ultrafine polymeric NFs with controlled dimensions and porosity to mimic ECM, was used^22,32^. It is also an ecofriendly and cost-effective technique that can be used easily to produce NFs on large scale. Producing well-organized highly porous wound dressings is essential in mimicking the nature of ECM^33^.

Consequently, these NFs-based wound dressings will help in increasing cell adhesion, proliferation, reorganization, and tissue regeneration. It will also help in facile permeation of oxygen and blood supply within the regenerating tissues for efficient healing^34–38^. This returns back to the resemblance of their dimensions and features to those of the natural collagen fibrils abundant in the human skin tissues^39,40^.

Many polymers have been approved by FDA for various biomedical applications including: (i) drug delivery such as anticancer, immunotherapy^24,41–45^, etc., (ii) tissue engineering^21,29,46^ and (iii) wound healing^21,29^ owing to their facile formulations, biocompatibility, biodegradability, etc. Many polymers have been used in fabricating NFs either natural-based such as chitosan, gelatin and collagen, or synthetic such as polyvinyl alcohol, polycaprolactone and others. Although most of naturally-based polymers are bioactive, they lack proper mechanical strength which negatively affects their durability. On the other hand, synthetic polymers have good mechanical properties but may show limited biocompatibility and biodegeradability^47^. Polylactic acid (PLLA), an FDA approved biopolymer, is characterized by being naturally derived and also possess reported high mechanical properties^27^, and it has been used in fabrication of NFs mats for the purpose of drug delivery and tissue engineering. This returns back to its facile spinnability, good biodegradability, and biocompatibility. Besides, PLLA is considered a unique biopolymer as it has good mechanical properties when compared to other biopolymers such as chitosan, gelatin and collagen. Good mechanical properties are desired in fabrication of durable wound dressings that could withstand long treatment duration as diabetic wounds. In addition, PLLA has been reported to enhance cells proliferation and growth factors signaling owing to its nanoporous structure and mechanical properties that resemble those of natural collagen fibers in ECM^48,49^.

Carbopol^®^ is a member of the polyacrylic acid polymers with a high swellability and could act as a mucoadhesive layer. This would help in the hemostatic phase. In addition, carbopol, as an acidic water soluble polymer, could help in neutralization of the high alkalinity of the diabetic wounds, thus preventing probability of microbial infection^50^.

In active wound dressings, some drugs, bioactive materials or growth factors are usually used to enhance the effeciency of the dressing in wound treatment and healing. Several drugs have been reported for their enhanced wound healing activity through different mechanisms of action. For instance, phenytoin (PHN) has been reported for its ability to decrease inflammation and edema through reducing the amount of exudates and polymorphonuclear nucleocytes (PMNL) within the damaged tissues. It is also capable of increasing the rate of fibroblast and myofibroblasts proliferation, tissue granulation and nerve regeneration. Finally, PHN has an inhibitory effect on collagenase enzyme which helps in increasing collagen deposition^11,51–53^.

Sildenafil citrate (SILD) is another promising drug for tissue regeneration due to its reported high ability of enhancing angiogenesis and vascularization within the damaged tissues. SILD has an inhibitory effect on phosphodiesterase II enzyme that breaks cGMP down. This in turn leads to an increase in nitric oxide (NO) gas production that stimulates formation of new blood vessels as well as dilatation of old ones within the regenerating tissues^8,9,54^.

Finally, simvastatin (SIM) has been reported for its ability to enhance wound healing process in diabetic wounds through stimulation of impaired lymphangiogenesis in diabetic patients. Lymph vessels are responsible for the drainage of protein-rich lymph from ECM, thus maintaining the normal tissue pressure and allowing the immune response to proceed normally. This also helps in decreasing the inflammation and consequently the probability of microbial infection^6,7,55,56^.

**Figure 1:**
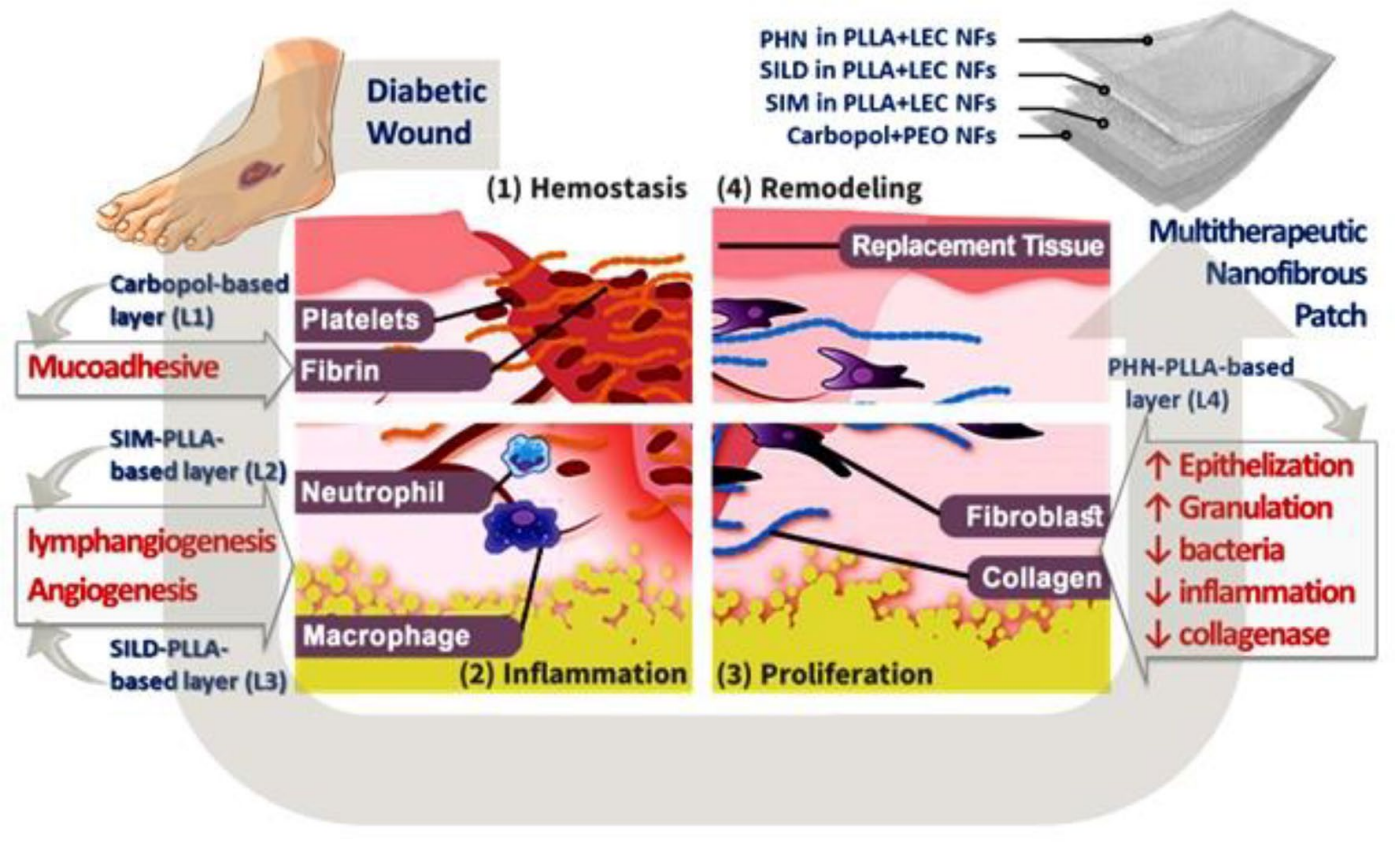
Schematic representation of patch design and function of each element.

Therefore, this study aims at designing a multilayered wound patch with a multifunctional strategy for efficient healing of complicated wounds like diabetic ulcer. It was designed in such a way so that each layer can perform a specific function to overcome the various problems associated with the diabetic wounds (Fig. 1).

For example, the first NFs layer is composed of carbopol and polyethylene oxide (PEO) blend. Carbopol, an acidic polymer, is responsible for decreasing the alkalinity of the diabetic wound thus inhibiting the growth of microorganisms. In addition, both carbopol and PEO are highly hydrophilic materials so they can swell and act as a mucoadhesive layer causing hemostasis.

The second NFs layer is made of PLLA/lecithin (LEC) and loaded with SIM that can overcome the issue of impaired lymphianogenesis in diabetic wounds, thus help in decreasing inflammation and edema. The third NFs layer is composed of PLLA/LEC and incorporating SILD to stimulate angiogenesis as well as vasodilatation of the old ones within the regenerating tissues. The fourth layer is composed of PLLA/LEC NFs containing PHN to enhance fibroblasts proliferation, tissue granulation and nerve regeneration.

These layers were designed and well-organized in accordance to the stages of the healing process. In other words, carbopol is needed at the beginning to decrease the alkalinity of the diabetic wounds, thus inhibit microorganisms’ growth. Then, comes SIM’s rule to stimulate lymphangiogenesis and decreases inflammation. SILD then comes after SIM to start increasing the blood supply and consequently nutrients to the regenerating tissues. Finally, PHN’s role is stimulating cells proliferation to accomplish proper wound healing.

## Results

### Characterization of NFs layers and multilayer patches

**Figure 2:**
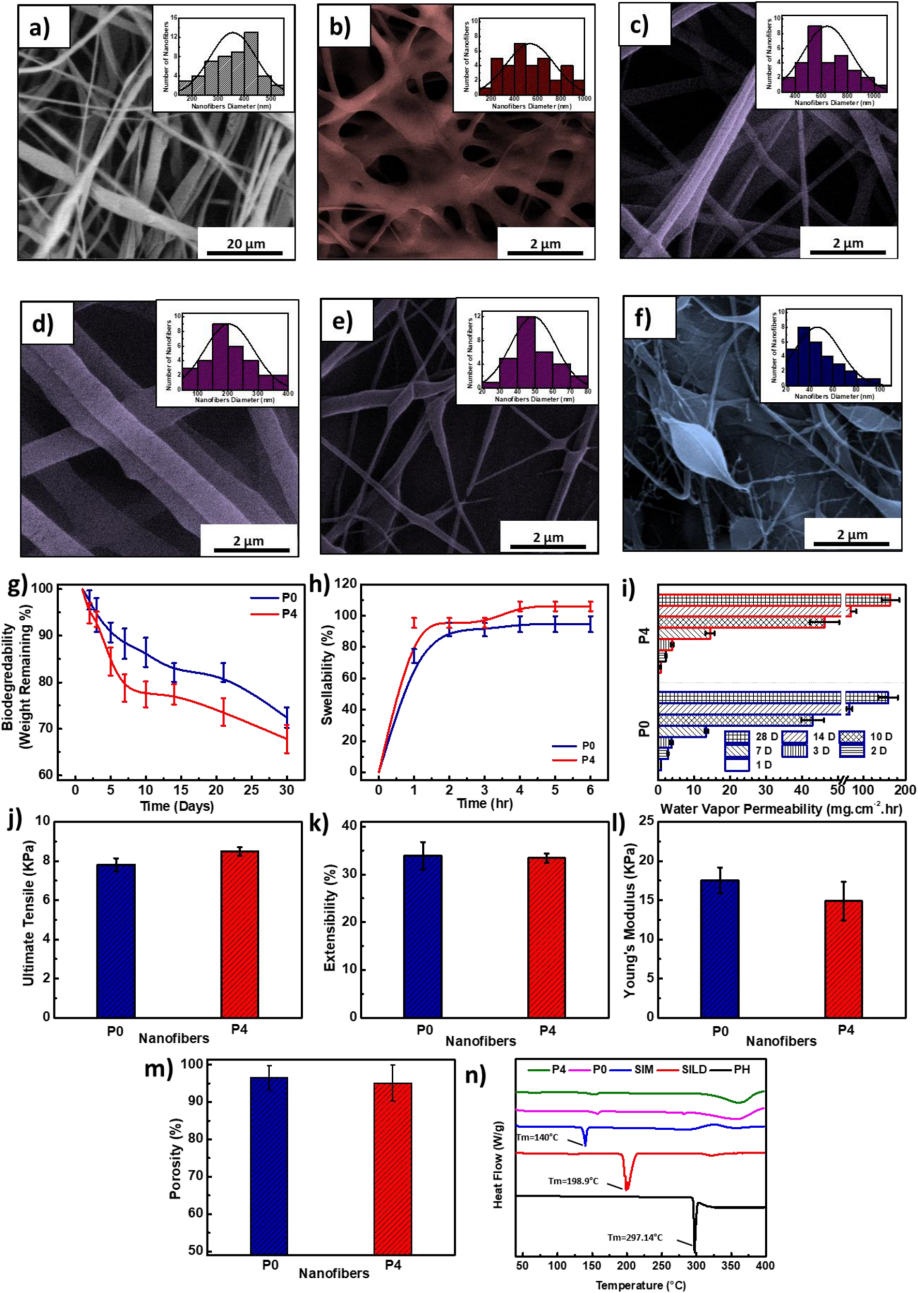
Morphological, physical and mechanical studies of NFs patches: SEM images of **(a)** PLLA/LEC NFs, and **(b–f)** carbopol/PEO NFs in the ratios of; **(b)** (0: 100), **(c)** (25: 75), **(d)** (50: 50), (**e**) (75: 25), and **(f)** (100: 0), **(g)** biodegradability, **(h)** swellability, **(i)** water vapor permeability (WVP), **(j)** ultimate tensile, **(k)** extensibility percentage, **(l)** young’s modulus and **(m)** porosity, and **(n)** DSC. Data are represented as mean ± SD (*p < 0.05, **p < 0.01, ***p < 0.001, ****p < 0.0001 and n = 3).

Different NFs layers of the wound patches were electrospun successfully and deposited simultaneously on the collector. The prepared NFs were uniform and randomly oriented network meshes that mimic the real structure of ECM. Figure 2a–f demonstrates the morphology of both PLLA/LEC NFs and carbopol/PEO NFs using SEM imaging. Figure 2a shows that the PLLA/LEC NFs possess firm porous structure that could be a base for fabrication of scaffolds. ImageJ analysis showed that the average diameter of the NFs is 709 ± 178 nm. Figure 2b–f show trials of L1 preparation through electrospinning of carbopol/PEO blend solution in five different ratios. Generally, increasing the carbopol ratio within the blend solution led to a gradual decrease in the NFs diameter, and it was also found that the NFs became more fragile and non-uniform.

Figure 2g–m illustrates the physical and mechanical characterization of both plain (P0), and drug-loaded wound patches. Figure 2g shows the plot of the calculated remaining weights (%) against certain time points. Both P0 and P4 lost about 25–30% of their weight within 30 days with P4 shows a slightly higher biodegradability rate than P0. Figure 2h shows the swelling profiles for both P0 and P4 patches. It was found that both NFs patches attained their maximum swellability in less than 4 h, with P4 demonstrated a slightly higher equilibrium swelling than P0. According to Fig. 2i, both P0 and P4 possess similar WVP.

Figure 2j–l demonstrates the ultimate tensile, extensibility % and young’s modulus, respectively for both P0 and P4. As apparent from the figure, there is no significant difference between the two patches regarding their mechanical properties. As per Fig. 2m, both P0 and P4 possess similar porosity.

Figure 2n depicts the DSC analysis of free drugs as well as P0 and P4. The thermograms revealed endothermic peaks at 297.14 °C, 198.9 °C and 140 °C owing to the melting points of PHN, SILD and SIM, respectively.

**Figure 3:**
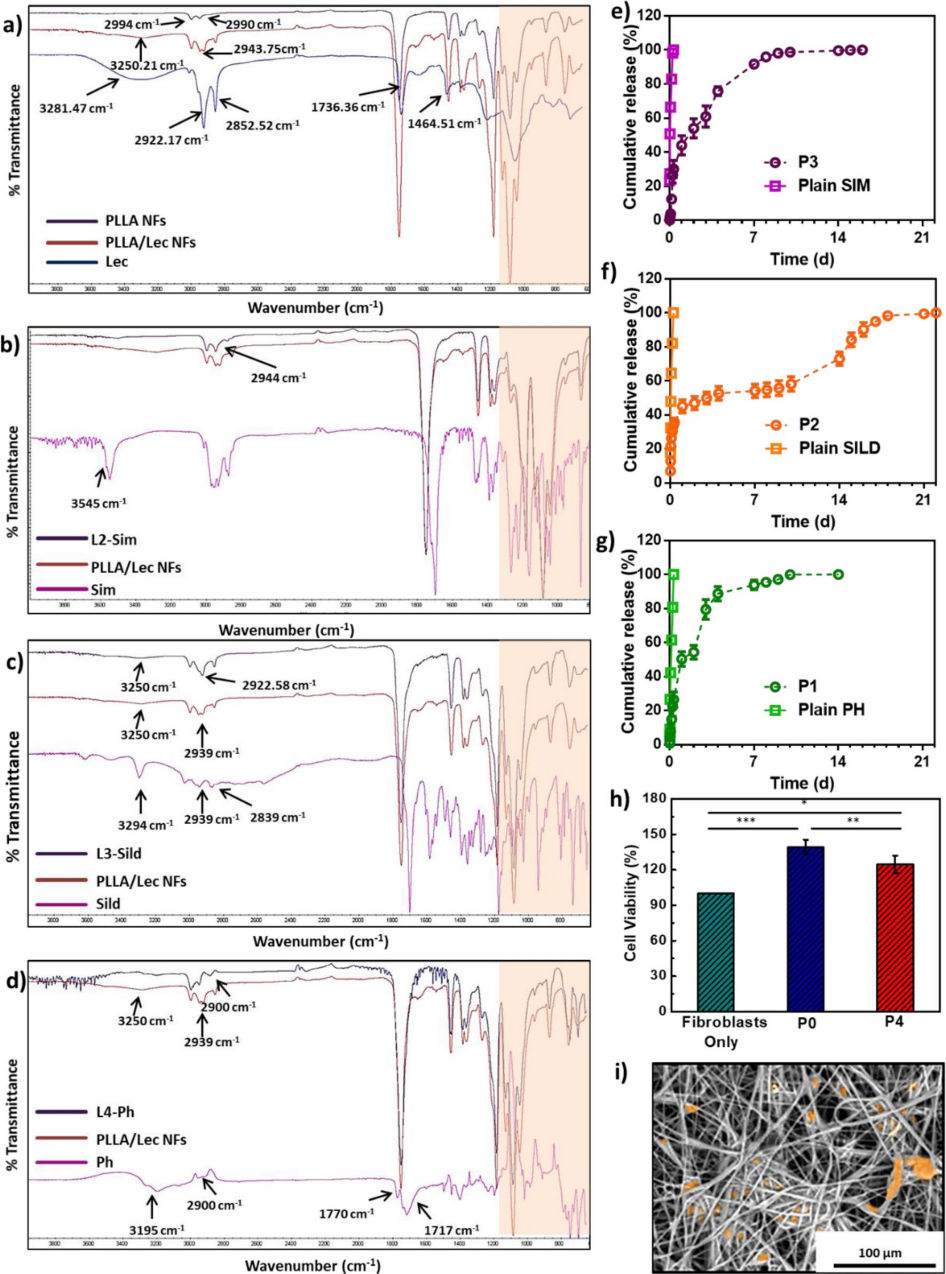
FTIR analysis: (**a**) Plain NFs, (**b**) L2-SIM, (**c**) L3-SILD, and (**d**) L4-PHN. *In-vitro* release of drugs-loaded NFs: (**e**) simvastatin (SIM) release profile, (**f**) sildenafil citrate (SILD) release profile, (**g**) phenytoin (PHN) release profile, In-vitro cell study of plain and drugs-loaded NFs: (**h**) cell viability, and (**i**) cell proliferation and attachment (fake coloring of cells on NFs). Data are represented as mean ± SD (*p < 0.05, **p < 0.01, ***p < 0.001, ****p < 0.0001 and n = 3).

Figure 3a demonstrates FTIR spectrum of PLLA/LEC NFs layer as compared to its components (PLLA and LEC). In addition, the FTIR spectra of L2-SIM, L3-SILD and L4-PHN are shown in Fig. 3b–d, respectively. FTIR analysis confirms the successful incorporation of the drugs within the NFs matrix through detecting their characteristic functional groups bands within the matrix of PLLA/LEC NFs.

PLLA NFs spectrum shows a needle-like peaks at 1170 cm^−1^ and 1147 cm^−1^ owing to the C–O and C=O stretchings, respectively along the polymer chains of PLLA. In addition, a mountainous triplet peak was also detected at 1025 cm^−1^, 1075 cm^−1^ and 1110 cm^−1^ that correspond to C–O stretching vibrations. This is attributed to the end-capped nature of the PLLA chains. Moreover, asymmetric and symmetric C–H stretchings were detected through two moderate peaks appearing at 2990 cm^−1^ and 2994 cm^−1^, respectively. All the obtained IR peaks of PLLA NFs are in agreement with that have been reported in the literature^48,57–59^.

The FTIR spectrum of LEC showed a broad moderate peak at around 3200 cm^−1^ owing to the stretching OH groups within the lipid structure. Absorption peaks of CH, CH_2_ and CH_3_ stretchings appeared in the region of 2800–3000 cm^−1^, however their bending bands appeared at around 1465 cm^−1^. Stretching of C=O bond of gycophosphatide ester linkage appeared at 1745 cm^-−1^. Additionally, characteristic peaks of P=O, C–O–C and P–O stretchings were recorded at 1190 cm^−1^, 1160 cm^−1^ and 1100 cm^−1^, respectively. These results are found to be in a good agreement with what has been found in previous work^60,61^.

The spectrum of SIM showed characteristic bands at 3545 cm^−1^ and 2900 cm^−1^ corresponding to O–H and C–H stretchings. In addition, two characteristic bands were detected at around 1700 cm^−1^ and 1160 cm^−1^ owing to the C=O and C–O stretchings^62^. The spectrum of free PHN depicted three characteristic peaks at 3195 cm^−1^, 1770 cm^−1^ and 1400 cm^−1^ owing to N–H, C=O and C–N, respectively^63^. The SILD spectrum showed three characteristic absorption bands at 1697 cm^−1^, 1550 cm^−1^ and 1150 cm^−1^ that correspond to aromatic C=C and C=O stretchings, and SO_2_, respectively. Characteristic peaks were recorded at 2893 cm^−1^ and 2939 cm^−1^ owing to saturated C–H stretchings in free SILD. On the other hand, peaks for unsaturated C–H stretchings were observed at 3029 cm^-1^. Finally, two peaks were noted at 3500 cm^−1^ and 3605 cm^−1^ which could be assigned to the secondary NH and OH stretchings, respectively^64^.

### In-vitro PHN/SILD/SIM cumulative release profiles and their kinetics modelling

Figure 3e–g shows the release profile of SIM, SILD and PHN from P3, P2 and P1, respectively. It was observed that 100% cumulative release of PHN, SILD and SIM from their wound patches were achieved after 11, 21 and 15 days, respectively. The release profiles of all patches were fitted to different kinetic models.

It was found that the suitable model for P3 (SIM release) was Makoid-Banakar model (r^2^ 0.98) with Kmb, n and k were found to be 7.2, 0.55 and 0.002, respectively. For P2 (SILD release), it also fitted Makoid-Banakar model (r^2^ 0.95) with Kmb, n and k of 22.04, 0.15 and − 0.001, respectively. For P1, the PHN was found to also follow Makoid-Banakar model (r^2^ 0.95) where the Kmb, n, and k attained 7.53, 0.57 and 0.002, respectively.

### Cell viability, adhesion and proliferation

Figure 3h shows the results of MTT assay carried out for P0 and P4 using human fibroblasts. It was observed that both patches possess high biocompatibility as the optical density of the fibroblasts with the tested patches exceeded that of those without any tested materials. There is a significant increase in cell viability of P0 (*p* < 0.001) and P4 (*p* < 0.05) when compared with control fibroblast cells. Figure 3i illustrates the adhesion of cells on the surface of P4 after being fixed and imaged using SEM.

### In-vivo assessments

**Figure 4:**
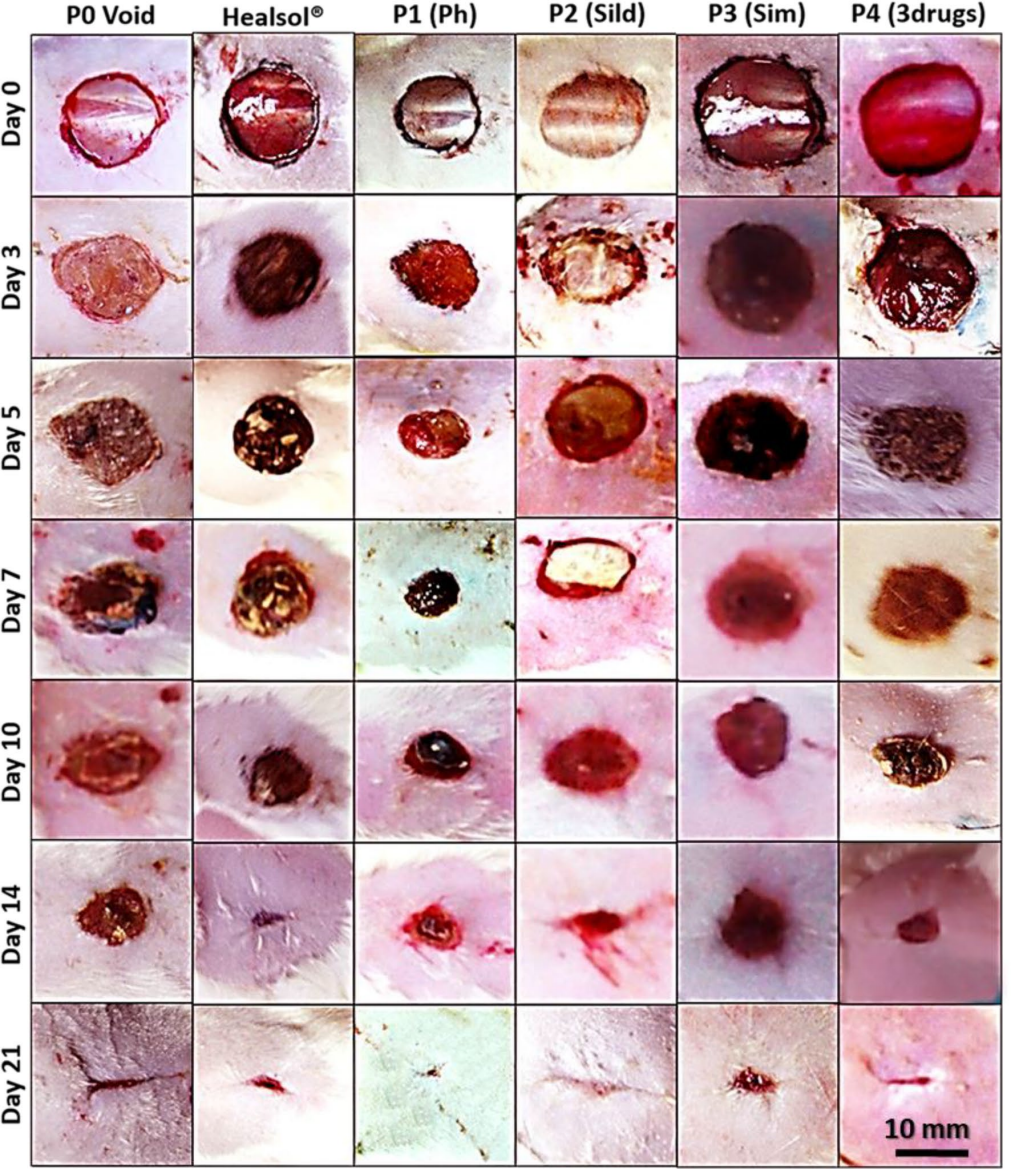
Visual inspection of wound healing effect of Healsol^®^ and the different developed patches over 21 days study.

Figure 4 summarizes the results of visual inspection of the healing progress of diabetic wounds treated with different patches. Healsol^®^ was used as a marketed product. Through the 21 days-study, it was observed that the different drug-loaded patches were successfully healed almost the entire wound area. It was also found that a single patch of triple drugs-loaded patches (P4) group was comparable in healing of wounds to multi-dose Healsol^®^ spray group. On the other hand, P0 and P2 groups showed slower rate of healing than other treated groups. Percentage of wound area was calculated for each group, and then the results were plotted as shown in Fig. 6b.

**Figure 5:**
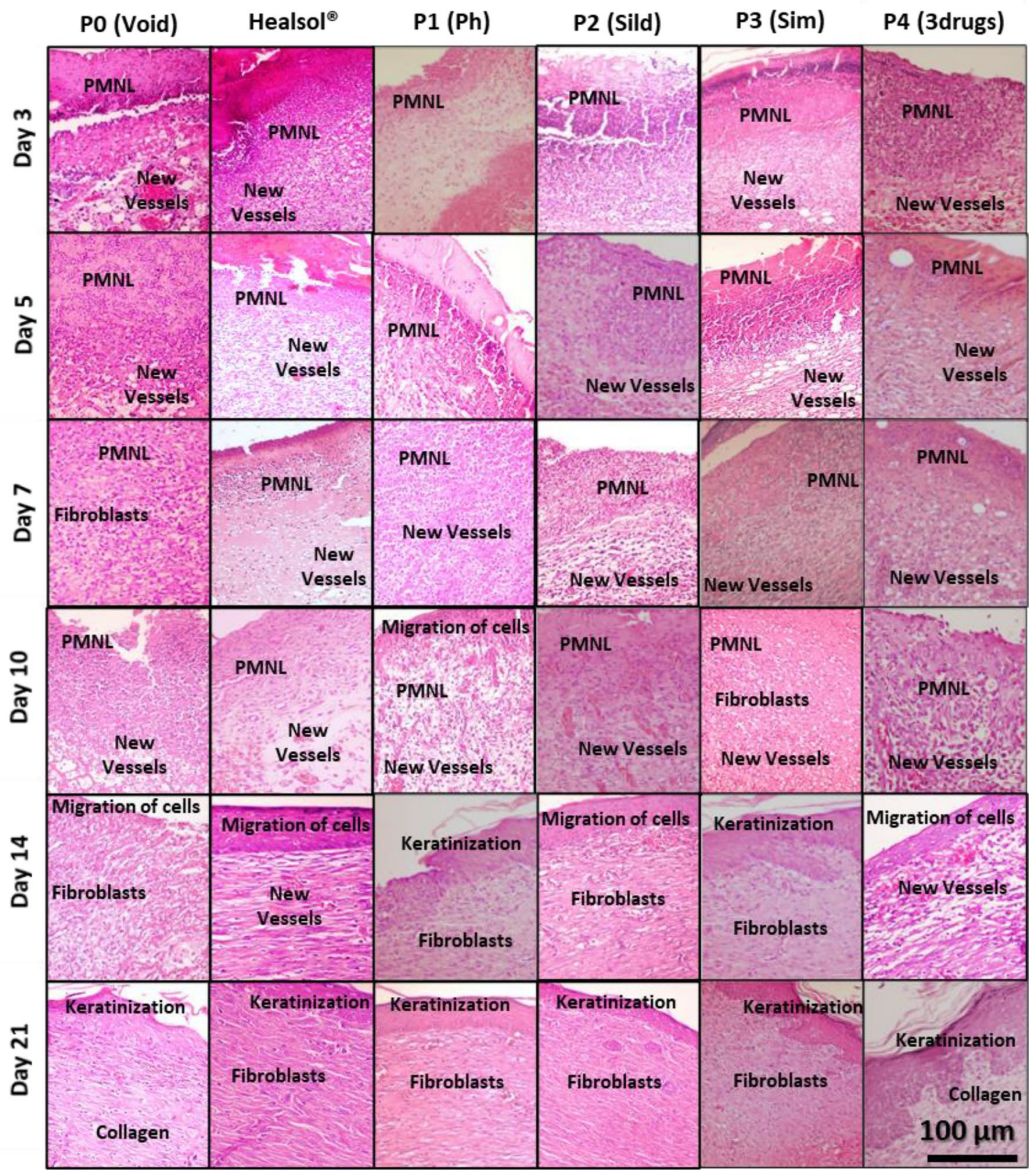
Histopathological examination using hematoxylin and eosin stains of specimen isolated from wound treated with Healsol^®^ and different developed NFs wound patches at different time points. (scale 200×).

**Figure 6:**
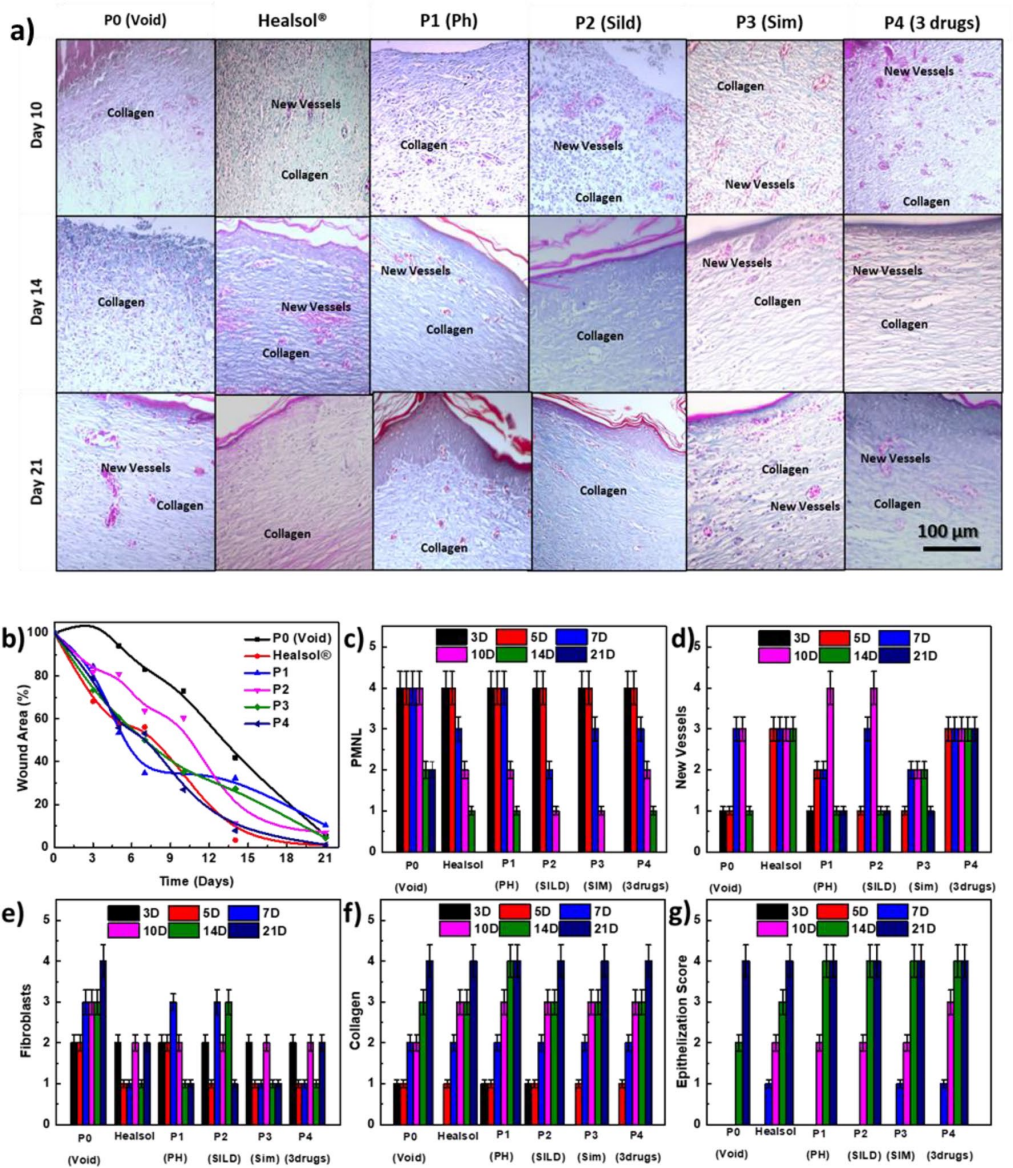
Histopathological examination for collagen deposition: (**a**) Masson’s trichrome stained specimens isolated from wounds treated with Healsol^®^ and different developed wound patches at different time points. (scale 200×). Evaluation of wound healing process in diabetic wound rat model: (**b**) wound area percentage, and scores of (**c**) PMNL, (**d**) new vessels epithelization, (**e**) fibroblasts, (**f**) collagen, and (**g**) epithelization.

Figures 5 and 6a show the histological investigation of wounds of all tested groups at the selected time points. Results after 3 days showed the formation of a thickened epidermal layer at the cut edge and rich with inflammatory cells (PMNL) in all of the tested groups (Fig. 6c). Slight neo-angiogenesis was observed in groups treated with P3 and P4 (Fig. 6d).

After 5 days, it was found that a fibrin net incorporating inflammatory cells (neutrophils, macrophages and lymphocytes) was formed in all animals (Fig. 6c). However, the epidermal layer formation was completely inhibited. Neo-angiogenesis started to appear in the rest of Healsol^®^, P0, P1 and P2 groups (Fig. 6d). In addition, mild fibroblasts proliferation (Fig. 6e) and collagen deposited (Fig. 6f) started to be observed.

After 7 days, the wound gap was intensely filled with both necrotic tissues and inflammatory cells (mainly neutrophils) in groups treated with Healsol, P0, P1 and P2 (Fig. 6c). However, groups treated with P3 and P4 showed minimal number of inflammatory cells and no necrotic tissue (Fig. 6c). Furthermore, granulation tissue composed of endothelial cells (Fig. 6g), fibroblasts (Fig. 6e) and non-organized collagen (Fig. 6f) was recorded at the bottom of wounds treated with P3 and P4.

After 10 days, groups treated with P1, P3 and P4 showed bridging the excision by epidermal basal layer (Fig. 6g). Other groups showed no significant changes except a decrease in the inflammatory cell intensity (Fig. 6c).

After 14 days, all treated groups except the groups treated with P0 showed almost a complete epidermal regeneration (Fig. 6g) with no inflammatory cells (Fig. 6c). Groups treated with P1, P2, P3 and P4 showed increased keratinocytes formation. In addition, their granulation tissues showed more collagen deposition (Fig. 6f) than endothelial cells and fibroblasts proliferation. All the treated groups demonstrated reorganized granulation tissue as well as a complete angiogenesis after 21 days. In addition, no inflammatory cells or necrotic tissues were observed.

## Discussion

The current study proposed a drug-eluting multilayered NFs patches for treatment of diabetic wounds. These patches were developed successfully using electrospinning technique. Two types of NFs were prepared; carbopol/PEO NFs and PLLA/LEC NFs. Due to the alkaline nature of diabetic wounds (pH 9), an acidic polymer, carbopol was selected to neutralize the pH.

Furthermore, carbopol has a good mucoadhesive property, which is important to fix the patch at the wound site. So, the carbopol NFs layer (L1) was allocated in the patches to face wound surface.

Generally, carbopol, a member of polyacrylic acid-based polymers, showed intense difficulty in electrospinning leading to formation of non-uniform and beaded NFs as shown in Fig. 2f, so, it has to be blended with another polymer to be easily electrospun. PEO was chosen to be blended with carbopol in different ratios as it is biodegradable FDA approved polymer that could be electrospun into uniform network meshes as shown in Fig. 2b. It was found that the highest possible ratio of carbopol in the blend that could be electrospinnable into uniform NFs is (1:1) as shown in Fig. 2d.

On the other hand, porosity is an important feature that should be available in any tissue scaffolds. It helps in allowing the perfusion of nutrients and oxygen during applying the patches and consequently, enhancing the rate of wound healing^65,66^. PLLA/LEC NFs were selected to act as porous backbone of the wound patch. PLLA/LEC NFs were obtained successfully via electrospinning using DCM as the solvent system.

DCM was found to be a convenient solvent as it has the lowest boiling point (39.2 °C) among other solvent systems (DMF and CHCl_3_). High volatility of DCM helped in obtaining uniform non-beaded highly porous NFs as shown in Fig. 2a. This makes the developed NFs a convenient scaffold that could mimic the porous structure of the ECM of epidermal layer. SIM, SILD and PHN were separately loaded into PLLA/LEC NFs to prepare L2, L3 and L4, respectively.

Multilayer patches were assembled as designed. After the fabrication of nanofibrous wound patches, physiochemical characterization (biodegradability, swellability, water vapor permeability and porosity) was done to investigate the effect of the drugs loaded within their matrices, and to test the capability of the prepared patches as scaffolds for skin regeneration.

It was found that the designed multiplayer patch (P0) has good biodegradability as it could lose around 30% of its weight within 30 days when placed in PBS buffer at pH 9 as shown in Fig. 2g. This is considered to be convenient for designing diabetic wound patches as the minimum treatment period of diabetic wound is around 3 weeks.

Thus, the patch can preserve its durability and withstand the long treatment period. Higher biodegradability may lead to losing the integrity of the patch leading to tearing before complete healing. In addition, it was found that loading drugs within the patch (P4) did not highly affect the degradability rate of the NFs matrices.

Drug-loaded patches showed slightly higher degradability than plain patches. This could be attributed to the presence of SILD, a hydrophilic drug, within the third layer of the patch that could increase the hydrophilicity of the NFs. Furthermore, it is concluded that the tested patches have good ability to absorb PBS buffer (pH 9) during the first hour and increase their swellability to exceeded 50% as shown in Fig. 2h. Afterwards, their swellability increased gradually during the second and third hour to reach almost 100%. It was found that swelling percentage reached a plateau after only three hours and continued during the following three hours confirming reaching maximum swelling. It was also found that drug-loaded patches showed a slight increase in their swelling ability compared to the plain ones. This is could be attributed to the hydrophilic nature of SILD abundant in the third layer of the patch that could facilitate the diffusion of PBS among the NFs structure.

WVP and porosity results (Fig. 2i, m) confirmed the high breathability and porosity of the prepared NFs patches, respectively. It was found that loading of drugs within the NFs matrices did not affect the porous structure of the prepared NFs negatively as there was no significance difference noted between P0 and P4. Results of both WVP and porosity also confirm the ability of using the prepared patches as wound dressings and scaffolding material. This conform with scaffold ideal characteristics as ability to enhance perfusion of nutrients and oxygen as well as proliferation of fibroblast owing to their high WVP and porosity, respectively^21,67–69^.

Mechanical properties (ultimate tensile, extensibility percentage and young’s modulus) of the wound patches were studied and plotted as shown in Fig. 2j–l to examine whether they were affected negatively due to high porosity of the patches or not. It was found that the obtained mechanical properties are within the acceptable range for handing during application and standing treatment time (more than 3 weeks) when compared with the other reported materials^70,71^. Moreover, it was found that the drugs loading did not affect the mechanical properties and consequently the integrity of the prepared patches. These results match the biodegedability profile. The physical and mechanical studies of the prepared patches confirmed their durability and robustness for application as wound dressings for more than three weeks.

Thermograms (Fig. 2n) of the three free drugs as well as P0 and P4 showed high stability against thermal degradation. The developed patches could withstand high temperature up to 120 °C without decomposition. Disappearance of the melting points of the three drugs in the thermogram of P4 confirms the homogenous distribution of the three drugs within the NFs matrices. Thermogram of P4 did not show any peak shifts when compared with that of P0, which confirms that the incorporation of the three drugs did not affect the crystalinity of the wound patches^72–75^.

FTIR spectra showed the successful incorporation of LEC within the PLLA NFs matrices. This is shown in Fig. 3a where, both characteristic peaks of PLLA and LEC were detected in the spectrum of PLLA/LEC NFs. Similarly, Fig. 3b–d showed the successful and homogenous incorporation of the three dugs, SIM, SILD and PHN, each in their corresponding PLLA/LEC NFs layer without noticeable interaction.

*In-vitro* drug release profiles of PHN, SILD and SIM, shown in Fig. 3e–g, illustrate the fast release of the three free drugs within 6–8 h only. However, the three drugs incorporated within the NFs wound patches, showed sustained release patterns.

For instance, around 25% of SIM was released steadily within the first 6 h from the second layer (P3). Afterwards; around 10% of SIM was released daily during the first week of the study. Around 100% was released after 11 days. On the other hand, SILD release was found to be little bit faster than SIM as more than 30% of the drug was released within the first 6 h from the third layer (P2). Then, SILD continued its release gradually to exceed 55% after 10 days. Afterwards, around 5–10% was released daily from the patch to reach almost 100% after 21 days. Similar to SIM, PHN showed around 25% release after the first 6–8 h followed by a gradual release to reach almost 55% after 2 days. PHN release kept going steadily till reaching 100% after 14 days. Generally, it was observed that around 20–25% of the three drugs were released out of the patch from their corresponding layers in the first 6–8 h. This could be related to the maximum swellability of the NFs that has been achieved in the same duration.

The steady release pattern of the three drugs could be linked to the gradual biodegradability that has been observed previously. The release profiles of all the patches were fitted to Makoid-Banakar model.

Both plain and drug-loaded patches (Fig. 3h) showed high biocompatibility when tested using human fibroblasts. It was observed that the cells density exceed the control (cells without materials). This proves the ability of the tested NFs to enhance the cell proliferation, which may be attributed to the highly porous structure of the prepared NFs. This also proves the biocompatibility of PHN, SILD and SIM^21,68^. Cell adhesion was confirmed after the cells have been fixed and imaged using SEM as shown in Fig. 3i. It has been found that fibroblasts (marked in an orange color) have been successfully adhered to the surface of the prepared patches. This proves that the prepared patches could be promising scaffolds to aid in wound healing according to the literature^18,76^.

*In-vivo* study was conducted to test our hypothesis of combing well-designed scaffold with multi drugs to target different wound healing stages. Visual inspection, histopathological investigation and collagen deposition of the diabetic wounds were summarized in Figs. 4, 5 and 6, respectively for 21 days on diabetic wound rat model. It was found that void patches were not capable of attaining complete closure of the wound area. In addition, scars were formed on the surface of the wounds.

Figure 6 demonstrates the scoring systems where PMNL (polymorphonuclear leucocytes) were observed to be more persistent in groups treated with either void patches or Healsol spray compared to the groups treated with drug-loaded patches. This explains the persistence of high inflammation in untreated groups owing to the absence of anti-inflammatory activity of some drugs such as PHN.

Histopathological images of wounds treated with void patches showed negligible fibroblasts proliferation, minimal vessel formation and collagen deposition throughout the study duration. Moreover, thick epithelization at the cut edges of the wounds indicated intense scars formation. On the other hand, it was observed that PHN-containing patches and formulas (Healsol^®^) showed noticeable enhanced wound closure and healing with no or minimal scars formation (patches and Healsol^®^, respectively). PHN has been reported to be capable of preventing scars formation through inhibiting bacterial accumulation and inflammation around the wound surfaces^21,77^. However, it was found that single PHN containing patches were more efficient in preventing scars formation than twice daily dose for 21 days of Healsol^®^. This could be attributed to abilities of NFs scaffold like porosity and swellability that could aid in absorbing the exudates and decreases inflammation. This was also confirmed by detecting more PMNL in wounds treated with Healsol^®^ compared to those treated with PHN containing patches. PHN has been also reported to enhance myofibroblasts and fibroblast proliferation that would consequently lead to acceleration of wound closure. This is attributed to the ability of PHN to promote growth factors activity and produce ECM^78^.

However, it was also found that the ability of PHN-containing patches to proliferate fibroblasts is more than Healsol^®^. This may return back to the porous structure of the NFs that mimics real ECM. Also, the high porosity increases the perfusion of nutrients that would help in increasing the rate of cells proliferation^36,79^. Finally, PHN-containing patches were able to help in collagen deposition due to the ability of PHN to reduce the activity of collagenase enzyme^77^.

Furthermore, it was observed that SILD-containing patches were successful to generate new blood vessels within the treated wounds starting the first week of treatment. This returns back to the reported ability of SILD to stimulate angiogenesis through production of nitric oxide gas. Nitric oxide gas acts as a vasodilator that enhances perfusion of blood and consequently nutrients to the tissues which aids in regeneration of damaged cells^8,9^. However, SILD showed no effect in decreasing inflammatory or necrotic cells like PHN. Moreover, SIM-containing patches were found to have good anti-inflammatory effect on the treated wounds. This returns back to the pleiotropic effect of SIM, the activity to enhance lymphianogenesis in diabetic wounds. This would help in the facile drainage of protein-rich lymph from ECM while maintaining normal tissue pressure allowing the immune system to perform normally^80,81^. It was also found that SIM has some angiogenesis effect, while minute cell proliferation activity.

The histological studies confirmed the high efficiency of the developed wound patches not only in wound healing but also in stimulating cells restoration and arrangement for proper wound healing. This was compared to the results obtained from the positive control group that has been carried out in our previous reported study, where the positive control group showed some wound healing progress. However, they failed to show proper cells restoration and tissue regeneration^21^.

Finally, the wound patches loaded with three complementary drugs possess the collective ability to treat complicated wounds like diabetic one with enhanced fibroblasts proliferation, angiogenesis and lymphianogenesis without scars formation or bacterial infection.

## Materials and methods

### Materials

Polylactic acid (PLLA) was provided as a gift by Purac Company, Holland. Phenytoin (PHN), sildenafil citrate (SILD) and simvastatin (SIM) were provided as gifts from El-Nasr Pharmaceutical Company (Egypt), Medical Union Pharmaceutical Company (Cairo, Egypt) and Hikma Pharmaceutical Company (Egypt), respectively. Healsol (ACDIMA) was purchased in its commercial form as a spray composed of 2% phenytoin base in propellant mixture. Acetone, acetic acid, dichloromethane (DCM), absolute ethanol (99%) and lecithin were purchased from Thermofischer, Germany. Carbopol and polyethylene oxide (900,000) were purchased from Acros, USA. 3-(4,5-dimethylthiazol-2yl)-2,5-diphenyl tetrazolium bromide (MTT) assay kit was obtained from Sigma-Aldrich, China. All other reagents were of analytical grade and used as received.

### Preparation of individual NFs layers

#### Preparation of carbopol/PEO NFs layer

Carbopol and PEO were individually dissolved in absolute ethanol to prepare 3% (w/v) solutions. Then, carbopol/PEO mixture solutions were prepared with different ratios (0: 100), (25: 75), (50: 50), (75: 25) and (100: 0) for electrospinning. Each solution was electrospun individually to determine the most appropriate electrospinnable ratio. NFs were obtained using an electrospinner (NANON—O1A instrument, MECC, Japan) using a previously reported setup^21^. The spinning parameters were found to be 1 ml/h feed rate, 12–15 keV applied voltage, and 15 cm distance between spinneret and the collector. The humidity and temperature inside the electrospinner were recorded to be 30% and 32 °C, respectively.

#### Preparation of PLLA/LEC NFs

PLLA/LEC solution (10% w/v) was prepared by dissolving the required amount of PLLA in DCM till obtaining a clear solution followed by addition of LEC in 10:1 as PLLA:LEC ratio. The resulting solution was electrospun using the previously used above setup to obtain the corresponding NFs (L1). The spinning parameters were recorded to be 3–5 ml/h feed rate, 19–21 keV, and 15 cm distance between the tip of the spinneret and the collector. The recorded humidity and temperature inside the electrospinner were 50–55% and 32 °C, respectively.

#### Preparation of drug-loaded NFs layers

Three different PLLA/LEC NFs layers were prepared with each layer containing a different drug (SIM, SILD or PHN). Each drug was added individually to PLLA/LEC solutions, with maintain the drug: PLLA ratio at 1:10 (L2-SIM, L3-SILD and L4-PHN). For L2-SIM, the SIM was added directly to the PLLA/LEC solution. For L3-Sild, few drops of acetic acid were added to the PLLA/LEC solution to dissolve SILD. For L4-PHN, PHN was dissolved in the minimal amount of acetone before being added to the PLLA/LEC solution. The electrospinning parameters and conditions were similar to that used upon fabrication of the plain PLLA/LEC NFs layer.

### Preparation of multilayer wound patches

Different multilayer wound patches were fabricated using electrospinning at the previously mentioned parameters and conditions in order to investigate the effect of each of the individual layers as well as their synergistic effects when combined. Table 1 summarizes five different fabricated wound patches and their compositions.

**Table 1:** Composition of the different developed multilayer wound patches.

Patches	Layer 1 (L1)	Layer 2 (L2)		Layer 3 (L3)		Layer 4 (L4)	
	Carbopol/PEO NFs	PLLA/LEC NFs	SIM	PLLA/LEC NFs	SILD	PLLA/LEC NFs	PHN
P0	✓	✓		✓		✓	
P1	✓	✓		✓		✓	✓
P2	✓	✓		✓	✓	✓	
P3	✓	✓	✓	✓		✓	
P4	✓	✓	✓	✓	✓	✓	✓

### Characterization of NFs layers and patches

Morphological investigations of the fabricated NFs layers were carried out using SEM (Nova Nano SEM, FEI, USA) after being gold plated. Then, ImageJ software (U.S. National Institutes of Health, Bethesda, Maryland, USA) was used to analyze the dimensions and polydispersity index of the imaged NFs.

Physical characterization of the plain and drug(s)-loaded wound patches was performed in order to study their biodegradability, swellability, porosity and breathability (water vapor permeability “WVP”). The experiments were done according to previously reported protocols that are used to estimate the appropriateness of the material to be used as scaffold for tissue regeneration^67,68,82,83^.

In-vitro biodegradability and swellability of the tested patches were carried out in PBS (pH 9) to mimic the real pH of diabetic wounds. Universal testing machine (Epsilon, USA) was used to estimate the mechanical strength (ultimate tensile, % extensibility and young’s modulus) of different patches.

Fourier transform infrared spectroscopy (FTIR) was used to investigate the chemical composition of the free drugs, plain NFs and drug-loaded NFs layers. The different samples were examined using FTIR (Thermoscientific, USA) in the range of 600–4000 cm^−1^. Besides, differential scanning calorimetry (DSC; Q20, TA instruments, USA) was used to detect the thermal stability of the free drugs, plain NFs and drug loaded patches.

### In-vitro cumulative drug release study

The cumulative release profile of each drug was studied individually using the dialysis bag diffusion method. During the experiment, PBS (pH 9) and ethanol with the ratio of 1:1 were used as dissolution medium to mimic the environment of the diabetic wounds and maintaining sink condition.

Briefly, a fixed weight of P1, P2 and P3 (equivalent to 2 mg of the loaded drug) was placed individually in donor compartment as previously reported^21,24^. At certain time points, 2 ml aliquot was withdrawn and replaced with another equivalent volume of fresh dissolution medium. The concentrations of PHN, SILD and SIM in the withdrawn aliquots were detected using UV–Vis spectrophotometer (Thermoscientific, USA) at wavelengths of 256 nm, 293 nm and 236 nm, respectively. Finally, the cumulative release profile was determined using previously reported Equations^21,84^.

Different kinetic models were examined to find the best fitting release profile including zero order, first order, Higuchi, Korsmeyer–Peppas, Hixson-Crowell, Hopfenberg, Baker-Lonsdale, and Makoid-Banakar^21,84^. The release experiments were performed in triplicates.

### Cell viability, adhesion and proliferation

MTT assay was used to estimate the biocompatibility of both plain and drug(s)-loaded NFs patches according to the previously reported protocol; ISO 10993-5^85,86^. Optical density of viable human dermal fibroblasts was assessed in absence and presence of the tested patches at the wavelength of 595 nm. Then, the cell viability percentage was calculated using the previously reported Equation^87^.

For cell adhesion and proliferation, 1 ml of fibroblasts suspension (1 × 10^5^ count) was added on circular discs (10 mm diameter) of P4 patch in 24-well plate. The samples were incubated in CO_2_ incubator for 48 h, then formalin was added for 6 h to fix adhered cells on the surfaces of the circular patches. Afterwards, the patches were washed with ethanol three times then examined using SEM imaging.

### In-vivo assessments

Animal experiments were performed according to institutional ethical guidelines. Approval of the wound healing animal model study (2016) was obtained from the Animal Research Committee in Misr University for Science and Technology (MUST). A total of 108 male albino rats with an average weight of 150 ± 20 g were used for the experiment. The animals were kept in a well-ventilated house with free access to food and drinking water during the entire experimental period.

#### Induction of diabetes and animal grouping

Diabetes was induced at the beginning of the experiment in the rats using alloxan monohydrate with a dose of 150 mg/kg. The calculated dose was administered intraperitoneally. The mice were kept during the whole duration of study in alternative light (12 h)/ night (12 h) cycles at room temperature. The mice were provided with normal chow diet and water along the 21-day study duration. After 36 h of the induction, the rates were left fasting for 12 h before estimating their fasting blood glucose levels. Afterwards, the tail arteries of these animals were punctured to collect the blood, and their blood glucose levels were determined using Accu-check active glucometer. Rats that showed fasting blood glucose levels between 250 and 400 mg/dl were considered diabetic^88^. Diabetic rats were randomly allocated into 6 groups each consisting of 18 rats.

#### Wound induction and patch application

The animals were anesthetized using ketamine then their dorsal back hair was shaved using a depilatory cream. A biopsy puncher (13 mm diameter) was used to inducting circular full thickness wounds (13 mm diameter) on the dorsal side of each rat, where, wound size exceeds the critical wound size. The entire skin was removed to uncover the underneath muscles. Afterwards, each group was treated with one of the following: P0, Healsol^®^, P1, P2, P3 and P4 to examine their effect in diabetic wound healing model. For all patches, the first layer (L1) was facing wound area. Healsol^®^, a PHN-based spray available in the market, was used as a marketed product with twice daily dose and covered by gauze. The patches were applied once at the beginning of the experiment till the end, while Healsol was applied twice per day along the whole study.

#### Wound closure measurement

The wound area of each animal was photographed using a digital camera after fixed time points. ImageJ software was used to measure the wound area. Then, wound area percentage was calculated according to previously approved calculations^21^.

#### Histopathological studies

At certain time points, three rats were euthanized from each group. Their surrounding skin specimens and muscles including the wound area were isolated and dipped in 10% formalin saline buffer for fixation. Afterwards, the samples were trimmed off, washed and dehydrated in ascending grades of alcohol. The dehydrated specimens were embedded in paraffin blocks to be preserved and sectioned at 4–6 µm thickness.

The obtained tissue sections were deparaffinized using xylol and stained using hematoxylin and eosin (H&E) and Masson’s trichrome for histopathological examination through the electric light microscope at a power of 200X**.** Semi-quantitative scoring method was used to evaluate following histological processes and structures: reepithelization, PMNL (polymorphonuclear leucocytes), fibroblasts, new vessels formation, and new collagen deposition. Sections were given scores according to the scale: 0, 1, 2, 3, 4 as shown in Table 2^89^.

**Table 2:** Semi-quantitative scoring method table.

Scale	Epithelization	PMNL	Fibroblasts	New vessels	Collagen
0	Thickness of cut edges	Absent	Absent	Absent	Absent
1	Migration of cells (< 50%)	Mild ST	Mild ST	Mild-SCT	Minimal-GT
2	Migration of cells (≥ 50%)	Mild DL/GT	Mild-GT	Mild-GT	Mild-GT
3	Bridging the excision	Moderate DL/GT	Moderate-GT	Moderate-GT	Moderate-GT
4	Keratinization	Marked DL/GT	Marked-GT	Marked-GT	Marked-GT

*GT* granulation tissue, *ST* surrounding tissues (tissue out of GT), *DL* demarcation line, *SCT* subcutaneous tissue.

### Statistical methods

Experimental data were expressed as means ± standard deviation from three replicate experiments unless otherwise indicated. Significant differences were examined by one-way analysis of variance (ANOVA) followed by Tukey’s post-hoc tests (*p < 0.05, **p < 0.01, ***p < 0.001 and ****p < 0.0001) using the GraphPad Prism Software version 8.4.3 (Dotmatics, Boston, USA).

### Ethical approval

Animal experiments were performed according to institutional ethical guidelines and in accordance with ARRIVE guidelines. Approval of the wound healing animal model study was obtained on 21 November, 2016 from the Scientific Research Ethical Committee, Misr University for Science and Technology (MUST).

## Conclusion

In conclusion, PLLA/LEC solution was electrospun successfully into highly porous and uniform NFs mats to be used as the basic matrix for the multilayer wound patch. PHN, SILD and SIM were individually incorporated into PLLA/LEC NFs porous layers, each in a separate layer to target a specific wound healing phase. It is concluded that the prepared multilayered wound NFs patches could act as a multifunctional scaffold for enhanced skin regeneration and wound healing. This is due to the presence of various successive layers that could act in harmony with each other performing a distinctive function in the wound healing process. For instance, starting from the side facing the wound, the first layer containing carbopol and PEO, act as a mucoadhesive layer as well as a neutralizing agent for the high alkaline pH of the diabetic wounds. The second layer containing SIM stimulates the impaired lymphianogenesis within the diabetic wounds to decrease inflammation and exudates. Third layer incorporating SILD stimulates angiogenesis thus increasing the blood supply and consequently nutrients to the damaged tissues. Finally, the fourth layer containing PHN stimulates cell proliferation, collagen deposition and scars inhibition.

The patches were fully characterized before being tested through both in-vitro and in-vivo studies.

TEM confirmed the successful formation of uniform NFs of PLLA-based layers as well as carbapol/PEO NFs. It was found that the highest possible ratio of carbapol/PEO that can produce uniform NFs is 1:1. Furthermore, physicochemical and mechanical studies revealed that incorporation of drugs within PLLA-based matrices did not significantly destroy the biodegradability, swellability, porosity and mechanical features of the NFs. This confirms the reliability of the produced NFs to produce durable wound patches that can last for the whole time of treatment. FTIR spectra confirmed the successful fabrication of PLLA/Lec NFs. Furthermore, incorporation of the three drugs, each in its layer, was confirmed through the corresponding FTIR spectra. In vitro release study showed that both PHN and SIM were released in a controlled manner within 14 days, while SILD was completely released within 21 days. This confirms the ability of the developed NFs to act as a sustained release drug carrier.

Through the in vivo and histopathological experiments, drug-loaded wound patches demonstrated their high effeciency to stimulate wound healing as well cells re-organization and restoration compared to the void patches and commercial Healsol spray. It was found that almost a complete healing with proper tissue regeneration were accomplished within the 21-day study. Therefore, this developed multilayered NFs-based patches could be promising for appropriate and integrated wound healing processes.

## Data Availability

All data generated or analyzed during this study are included in this published article, and other data will be available on a reasonable request.

## Abbreviations

NFs: Nanofibers
PLLA: Polylactic acid
LEC: Lecithin
PLLA/LEC NFs: PLLA NFs containing 10% (w/w) of lecithin
PHN: Phenytoin
SILD: Sildenafil citrate
SIM: Simvastatin
PEO: Polyethylene oxide
L1: Carbopol/PEO NFs
L2-SIM: PLLA/LEC NFs containing 10% (w/w) of simvastatin
L3-SILD: PLLA/LEC NFs containing 10% (w/w) of sildenafil citrate
L4-PHN: PLLA/LEC NFs containing 10% (w/w) of phenytoin

## References

[1] Egede LE, Ellis C (2010). Diabetes and depression: Global perspectives. Diabetes Res. Clin. Pract..

[2] Lv H (2022). Electrospun chitosan-polyvinyl alcohol nanofiber dressings loaded with bioactive ursolic acid promoting diabetic wound healing. Nanomaterials.

[3] Pecoraro RE, Reiber GE, Burgess EM (1990). Pathways to diabetic limb amputation. Basis for prevention. Diabetes Care.

[4] Zhang P (2016). Global epidemiology of diabetic foot ulceration: a systematic review and meta-analysis Global epidemiology of diabetic foot ulceration: A systematic review and meta-analysis. Ann. Med..

[5] Liang Y, He J, Guo B (2021). Functional hydrogels as wound dressing to enhance wound healing. ACS Nano.

[6] Saaristo A (2006). Vascular endothelial growth factor-C accelerates diabetic wound healing. Am. J. Pathol..

[7] Maruyama K (2007). Decreased macrophage number and activation lead to reduced lymphatic vessel formation and contribute to impaired diabetic wound healing. Am. J. Pathol..

[8] Gürsoy K (2014). Effect of topically applied sildenafil citrate on wound healing: experimental study. Bosn. J. Basic Med. Sci..

[9] Cakmak E (2014). Effect of sildenafil citrate on secondary healing in full thickness skin defects in experiment. Bratisl. Lek. Listy.

[10] Fadini GP (2010). The redox enzyme p66Shc contributes to diabetes and ischemia-induced delay in cutaneous wound healing. Diabetes.

[11] Lodha SC (1992). New application of an old drug: Topical phenytoin for burns. J. Burn Care Res..

[12] Ji RC (2005). Characteristics of lymphatic endothelial cells in physiological and pathological conditions. Histol. Histopathol..

[13] Sengupta M, Sengupta J, Banerjee P, Ghosh M, Paul S (2015). Healing effect of phenytoin on excisional wound in experimental albino rats. Muller J. Med. Sci. Res..

[14] Anstead GM, Hart LM, Sunahara JF, Liter ME (1996). Phenytoin in wound healing. Ann. Pharmacother..

[15] Alven S, Peter S, Mbese Z, Aderibigbe BA (2022). Polymer-based wound dressing materials loaded with bioactive agents: Potential materials for the treatment of diabetic wounds. Polym. Basel.

[16] Xie Z (2013). Dual growth factor releasing multi-functional nanofibers for wound healing. Acta Biomater..

[17] Paul W, Sharma C (2004). Chitosan and alginate wound dressings: a short review. Trends Biomater Artif Organs.

[18] El-sherbiny IM, Ali IH (2015). Eco-friendly electrospun polymeric nanofibers-based nanocomposites for wound healing and tissue engineering. Eco-Friendly Polymer Nanocomposites.

[19] Muzzarelli RAA (2009). Chitins and chitosans for the repair of wounded skin, nerve, cartilage and bone. Carbohydr. Polym..

[20] Zahedi P, Rezaeian I, Ranaei-Siadat S-O, Jafari S-H, Supaphol P (2010). A review on wound dressings with an emphasis on electrospun nanofibrous polymeric bandages. Polym. Adv. Technol..

[21] Ali IH, Khalil IA, El-Sherbiny IM (2016). Single-dose electrospun nanoparticles-in-nanofibers wound dressings with enhanced epithelialization, collagen deposition, and granulation properties. ACS Appl. Mater. Interfaces.

[22] Sun M, Chen S, Ling P, Ma J, Wu S (2022). Electrospun methacrylated gelatin/poly(L-lactic acid) nanofibrous hydrogel scaffolds for potential wound dressing application. Nanomaterials.

[23] Ullah S (2020). Reusability comparison of melt-blown vs nanofiber face mask filters for use in the coronavirus pandemic. ACS Appl. Nano Mater..

[24] Khalil IA, Ali IH, El-Sherbiny IM (2019). Noninvasive biodegradable nanoparticles-in-nanofibers single-dose ocular insert: In vitro, ex vivo and in vivo evaluation. Nanomedicine.

[25] Bakhit M (2020). Downside of face masks and possible mitigation strategies: A systemin review and meta-analysis. medRxiv.

[26] Shahriar SMS (2019). Electrospinning nanofibers for therapeutics delivery. Nanomaterials.

[27] Ali IH, Khalil IA, El-Sherbiny IM (2019). Phenytoin/sildenafil loaded poly(lactic acid) bilayer nanofibrous scaffolds for efficient orthopedics regeneration. Int. J. Biol. Macromol..

[28] Huang Z-M, Zhang Y-Z, Kotaki M, Ramakrishna S (2003). A review on polymer nanofibers by electrospinning and their applications in nanocomposites. Compos. Sci. Technol..

[29] Ali IH (2022). Antimicrobial and wound-healing activities of graphene-reinforced electrospun chitosan/gelatin nanofibrous nanocomposite scaffolds. ACS Omega.

[30] Keirouz A, Chung M, Kwon J, Fortunato G, Radacsi N (2020). 2D and 3D electrospinning technologies for the fabrication of nanofibrous scaffolds for skin tissue engineering: A review. Wiley Interdiscip. Rev. Nanomed. Nanobiotechnol..

[31] Zaarour B, Zhu L, Huang C, Jin X (2020). A mini review on the generation of crimped ultrathin fibers via electrospinning: Materials, strategies, and applications. Polym. Adv. Technol..

[32] Wu S (2022). Electrospun conductive nanofiber yarns for accelerating mesenchymal stem cells differentiation and maturation into Schwann cell-like cells under a combination of electrical stimulation and chemical induction. Acta Biomater..

[33] Liu J, Zhai H, Sun Y, Wu S, Chen S (2021). Developing high strength poly(L-lactic acid) nanofiber yarns for biomedical textile materials: A comparative study of novel nanofiber yarns and traditional microfiber yarns. Mater. Lett..

[34] Ma PX (2004). Scaffolds for tissue fabrication. Mater. Today.

[35] Teo WE, Ramakrishna S (2006). A review on electrospinning design and nanofibre assemblies. Nanotechnology.

[36] Murugan R, Ramakrishna S (2006). Nano-featured scaffolds for tissue engineering: a review of spinning methodologies. Tissue Eng..

[37] Sill TJ, Von Recum HA (2008). Electrospinning: Applications in drug delivery and tissue engineering. Biomaterials.

[38] Pham QP, Sharma U, Mikos AG (2006). Electrospinning of polymeric nanofibers for tissue engineering applications: A review. Tissue Eng..

[39] Wu S (2021). Combining electrospinning with hot drawing process to fabricate high performance poly (L-lactic acid) nanofiber yarns for advanced nanostructured bio-textiles. Biofabrication.

[40] Wu S (2022). State-of-the-art review of advanced electrospun nanofiber yarn-based textiles for biomedical applications. Appl. Mater. Today.

[41] Abd-Algaleel SA, Metwally AA, Abdel-Bar HM, Kassem DH, Hathout RM (2021). Synchronizing in silico, in vitro, and in vivo studies for the successful nose to brain delivery of an anticancer molecule. Mol. Pharm..

[42] Khater SE, El-khouly A, Abdel-Bar HM, Al-mahallawi AM, Ghorab DM (2021). Fluoxetine hydrochloride loaded lipid polymer hybrid nanoparticles showed possible efficiency against SARS-CoV-2 infection. Int. J. Pharm..

[43] Abdel-Bar HM, Osman R, Abdel-Reheem AY, Mortada N, Awad GAS (2016). Tunable biodegradable nanocomposite hydrogel for improved cisplatin efficacy on HCT-116 colorectal cancer cells and decreased toxicity in rats. Biomacromol.

[44] Hao W (2020). 3D printing-based drug-loaded implanted prosthesis to prevent breast cancer recurrence post-conserving surgery. Asian J. Pharm. Sci..

[45] Abdel-Bar M (2021). An ‘eat me’ combinatory nano-formulation for systemic immunotherapy of solid tumors. Theranostics.

[46] Arida IA, Ali IH, Nasr M, El-Sherbiny IM (2021). Electrospun polymer-based nanofiber scaffolds for skin regeneration. J. Drug Deliv. Sci. Technol..

[47] Arida IA, Ali IH, Nasr M, El-Sherbiny IM (2021). Electrospun polymer-based nanofiber scaffolds for skin regeneration. J. Drug Deliv. Sci. Technol..

[48] Liu W, Dong Y, Liu D, Bai Y, Lu X (2018). Polylactic acid (PLA)/cellulose nanowhiskers (CNWs) composite nanofibers: Microstructural and properties analysis. J. Compos. Sci..

[49] Chen J (2011). PLLA-PEG-TCH-labeled bioactive molecule nanofibers for tissue engineering. Int. J. Nanomedicine.

[50] Zinovev EV, Ivakhniuk GK, Dadaian KA, Lagvilava TO (2014). Wound-healing effect of carbopol hydrogels in rats with alloxan diabetes model. Eksp. Klin. Farmakol..

[51] Bansal NK (1993). Comparison of topical phenytoin with normal saline in the treatment of chronic trophic ulcers in leprosy. Int. J. Dermatol..

[52] Yadav JK, Singhvi AM, Kumar N, Garg S (1993). Topical phenytoin in the treatment of split-thickness skin autograft donor sites: A comparative study with polyurethane membrane drape and conventional dressing. Burns.

[53] Menezes J, Rajendran A, Jacob AJ, Vaz M (1993). The use of topical phenytoin as an adjunct to immobilization in the treatment of trophic leprosy ulcers. Southeast Asian J. Trop. Med. Public Health.

[54] Shurbaji S (2021). Nitric oxide releasing hydrogel nanoparticles decreases epithelial cell injuries associated with airway reopening. Front. Bioeng. Biotechnol..

[55] Witte MH, Bernas MJ, Martin CP, Witte CL (2001). Lymphangiogenesis and lymphangiodysplasia: From molecular to clinical lymphology. Microsc. Res. Tech..

[56] Oliver G, Detmar M (2002). The rediscovery of the lymphatic system: old and new insights into the development and biological function of the lymphatic vasculature. Genes Dev..

[57] Chieng B (2013). Poly(lactic acid)/Poly(ethylene glycol) polymer nanocomposites: Effects of graphene nanoplatelets. Polymers (Basel)..

[58] Trang Mai TT (2012). A novel nanofiber Cur-loaded polylactic acid constructed by electrospinning. Adv. Nat. Sci. Nanosci. Nanotechnol..

[59] Sadeghi-Avalshahr AR, Khorsand-Ghayeni M, Nokhasteh S, Molavi AM, Sadeghi-Avalshahr M (2016). Physical and mechanical characterization of PLLA interference screws produced by two stage injection molding method. Prog. Biomater..

[60] Navneet N (2012). Examine the presence of chemical constituents in tecomella undulata. Int. J. Res. Pharm. Sci..

[61] El-Shattory Y, El-Magoli SB, Abu-Ria SH, Megahed MG (1999). Chemical and physical characteristics of local lecithin in comparison with some other food emulsifiers. Grasas Aceites.

[62] Singh H, Philip B, Pathak K (2012). Preparation, characterization and pharmacodynamic evaluation of fused dispersions of simvastatin using PEO-PPO block copolymer. Iran. J. Pharm. Res. IJPR.

[63] Kadam A, Jangam S, Oswal R (2011). Application of green chemistry principle in synthesis of phenytoin and its biogical evaluation as anticonvulsant agents. E-J. Chem..

[64] Podder AK, Chakrobarty JK, Faroque ABM (2014). Qualitative and quantitative analysis of sildenafil in traditional medicines and dietary supplements. Asian J. Pharm. Clin. Res..

[65] Jiankang H (2009). Preparation of chitosan-gelatin hybrid scaffolds with well-organized microstructures for hepatic tissue engineering. Acta Biomater..

[66] Nazarov R, Jin H-J, Kaplan DL (2004). Porous 3-D scaffolds from regenerated silk fibroin. Biomacromol.

[67] Sharma C, Dinda AK, Mishra NC (2013). Fabrication and characterization of natural origin chitosan- gelatin-alginate composite scaffold by foaming method without using surfactant. J. Appl. Polym. Sci..

[68] Vargas EAT, Do Vale Baracho NC, De Brito J, De Queiroz AAA (2010). Hyperbranched polyglycerol electrospun nanofibers for wound dressing applications. Acta Biomater..

[69] Lu L (2013). Silver nanoparticle/chitosan oligosaccharide/poly(vinyl alcohol) nanofibers as wound dressings: A preclinical study. Int. J. Nanomedicine.

[70] Sun K (2011). Preparations, properties and applications of chitosan based nanofibers fabricated by electrospinning. Polym. Lett..

[71] Liu Y, Kim H-I (2012). Characterization and antibacterial properties of genipin-crosslinked chitosan/poly(ethylene glycol)/ZnO/Ag nanocomposites. Carbohydr. Polym..

[72] Thakur R, Gupta RB (2006). Formation of phenytoin nanoparticles using rapid expansion of supercritical solution with solid cosolvent (RESS-SC) process. Int. J. Pharm..

[73] Vijay N, Murowchick JB, Youan B-BC (2010). Thermodynamics of drug nanoencapsulation: Case study of phenytoin-poly (D, L-lactide) nanocarrier. Curr. Drug Deliv..

[74] Ghasemian E (2013). Preparation, characterization and optimization of sildenafil citrate loaded PLGA nanoparticles by statistical factorial design. DARU J. Pharm. Sci..

[75] Castro AGB (2018). Incorporation of simvastatin in PLLA membranes for guided bone regeneration: Effect of thermal treatment on simvastatin release. RSC Adv..

[76] Zhong SP, Zhang YZ, Lim CT (2010). Tissue scaffolds for skin wound healing and dermal reconstruction. Wiley Interdiscip. Rev. Nanomed. Nanobiotechnol..

[77] Rezaeian I (2015). Drug release, cell adhesion and wound healing evaluations of electrospun carboxymethyl chitosan/polyethylene oxide nanofibres containing phenytoin sodium and vitamin C. IET Nanobiotechnol..

[78] Hasamnis A, Mohanty B, Patil S (2010). Evaluation of wound healing effect of topical phenytoin on excisional wound in albino rats. J. Young Pharm..

[79] Khang D, Carpenter J, Chun YW, Pareta R, Webster TJ (2010). Nanotechnology for regenerative medicine. Biomed. Microdevices.

[80] Cell S, Therapy C (2016). Pleiotropic effects of simvastatin on wound healing in diabetic mice. J. Med. Assoc. Thai.

[81] Asai J, Takenaka H, Hirakawa S (2012). Topical simvastatin accelerates wound healing in diabetes by enhancing angiogenesis and lymphangiogenesis. AJPA.

[82] Li CW (2013). Silver nanoparticle/chitosan oligosaccharide/poly(vinyl alcohol) nanofibers as wound dressings: A preclinical study. Int. J. Nanomedicine.

[83] Sreedhara S, Tata N (2013). A novel method for measurement of porosity in nanofiber mat using pycnometer in filtration. J. Eng. Fabr. Fibers..

[84] Costa P, Sousa Lobo JM (2001). Modeling and comparison of dissolution profiles. Eur. J. Pharm. Sci..

[85] Senthilraja P, Kathiresan K (2015). In vitro cytotoxicity MTT assay in Vero, HepG2 and MCF -7 cell lines study of Marine Yeast. J. Appl. Pharm. Sci..

[86] Hefnawy A, Khalil IA, El-Sherbiny IM (2017). Facile development of nanocomplex-in-nanoparticles for enhanced loading and selective delivery of doxorubicin to brain. Nanomedicine.

[87] Askari, P., Zahedi, P. & Rezaeian, I. Three-layered electrospun PVA/PCL/PVA nanofibrous mats containing tetracycline hydrochloride and phenytoin sodium: A case study on sustained control release, antibacterial, and cell culture properties. *J. Appl. Polym. Sci.* **133** (2016).

[88] Ajiboye BO, Oloyede HOB, Salawu MO (2018). Antihyperglycemic and antidyslipidemic activity of *Musa paradisiaca* -based diet in alloxan-induced diabetic rats. Food Sci. Nutr..

[89] Suvarna SK, Layton C, Bancroft JD (2018). Bancroft’s Theory and Practice of Histological Techniques.

